# Punching Shear Failure Analysis of Reinforced Concrete Slabs under Close-in Explosion

**DOI:** 10.3390/ma16186339

**Published:** 2023-09-21

**Authors:** Sheng Liu, Xiangyun Xu, Bukui Zhou, Kezhi Yang

**Affiliations:** Institute of Defense Engineering, AMS, PLA, Beijng 100850, China; lius950920@163.com (S.L.); zbk751225@sina.com (B.Z.); ykezhi@sina.com (K.Y.)

**Keywords:** reinforced concrete slab, explosion load, punching shear failure, twin shear stress yield criterion

## Abstract

The susceptibility of reinforced concrete (RC) slabs to punching shear failure is heightened when subjected to close-in explosion loads, leading to a wider range of damage caused by the resultant leakage shock wave through the punching hole. Consequently, it is crucial to analyze the conditions for punching shear failure and the size of the punching hole in RC slabs. This study investigates the punching shear failure of RC slabs under close-in explosions through numerical simulation and theoretical analysis. Initially, a finite element model is developed to examine the distribution of the explosion load on the slab’s surface. Subsequently, the critical explosion load for punching shear failure is determined using a damage criterion specific to RC slabs. Additionally, a calculation model based on the twin shear stress yield criterion is established to predict the punching hole’s radius. To validate the accuracy of this method, a comparison is conducted with existing test results.

## 1. Introduction

In light of the recent rise in explosion incidents caused by both terrorist attacks and accidental explosions, ensuring the anti-explosion safety of significant economic facilities and everyday civilian structures has presented challenges and requirements for engineering and academic communities [1,2,3,4,5,6]. Due to their relatively thin section thickness and expansive area of exposure to pressure, reinforced concrete (RC) slabs are particularly vulnerable to severe damage under explosion loads. Such damage can lead to the propagation of explosive shock waves over a wider range, resulting in significant property losses and casualties. Therefore, there has been extensive research on the dynamic response analysis and failure modes of RC slabs under explosion loads.

In recent years, notable achievements have been made in this field. For instance, Peng et al. [7] conducted an experimental study on the dynamic response of RC slabs under long-duration near-plane explosion loads, analyzing and summarizing the damage characteristics of RC slabs in such scenarios. Wang et al. [8] performed a series of experiments to examine the impact of scale on the degree of damage to RC slabs under close-in explosions, establishing two empirical formulas based on the experimental results to account for the scale influence. Feng et al. [9] investigated the dynamic response of RC slabs with reinforcement ratios ranging from 0.9% to 1.8% under close-in explosions, observing that increased reinforcement ratios led to reduced deflection and enhanced anti-explosion performance of the slabs. Kumar et al. [10] explored the failure mode and degree of damage in RC slabs through experiments, revealing that changes in explosion distance had a more significant effect on the damage degree than variations in charge weight. Shi et al. [11] studied the local damage of RC slabs under close-in explosion loads, analyzing the detailed influence of charge weight on the mass, size, and velocity of fragments. Wang et al. [12] conducted a series of tests to investigate the dynamic response and failure modes of RC slabs under close-in explosions, establishing damage criteria corresponding to different levels of damage based on the test results. Lu and Silva [13,14] proposed a procedure for predicting the appropriate explosive charge weight and standoff distance, considering a desired level of damage in RC slabs, and a series of tests verified its applicability. Given the costs and time associated with explosion tests, advanced numerical methods such as smoothed particle hydrodynamics and finite element methods have become widely utilized in the study of the dynamic response of RC slabs under explosion [15,16,17,18,19,20,21]. Yankelevsky et al. [22] studied the failure characteristics and damage patterns of the column-supported RC slab subjected to impulsive loading through numerical simulation, observing that damage was mainly concentrated at the connection between the slab and column. Design guidelines [23,24] have also been developed, providing information on the level of damage in RC slabs for different combinations of explosive charge weights and standoff distances.

The dynamic response and damage modes of RC slabs subjected to blast loads are primarily influenced by factors such as explosive charge weight, standoff distance, and material properties [25]. Under large standoff distances or lighter explosive charge weights, RC slabs tend to experience flexural damage. However, as the explosive charge weight increases and the standoff distance decreases, the failure mode of RC slabs gradually transits from global flexural damage to local punching shear failure. Recent years have seen considerable research on the factors contributing to the damage of RC slabs under explosion loads. Empirical formulas based on explosion tests have been developed to assess whether an RC member would sustain damage when subjected to an explosion load [26,27,28]. These formulas consider parameters such as the member’s thickness, concrete compressive strength, and explosion load parameters (explosive charge weight and standoff distance). While these formulas are simple, they cannot accurately differentiate between damage modes such as flexural damage and punching damage in RC slabs. Shaaban et al. [29] conducted a series of experiments to study the influence of different premature loads on the punching resistance of RC slabs, comparing the accuracy of the ultimate punching shear strength equations of RC slabs in different codes. Mottoni [30] introduced the Critical Shear Crack Theory (CSCT), which is based on punching capacity models to address cases involving impulsive behavior. The CSCT defines the bearing capacity as a function of slab deformation, indicating the relationship between concrete strain and critical shear cracks. Kinematics and constitutive equations are established to describe stress transfer along the critical crack through aggregate interlocking and tensile stress in the concrete. Sagaseta et al. [31] adapted the CSCT to analyze the local damage of RC slabs under close-in explosion loads, developing a maximum punching shear demand equation based on the dynamic equilibrium between pressure and inertial forces when local punching shear damage occurs in RC slabs. However, current research on the punching shear failure of RC slabs under explosion loads has primarily focused on failure conditions, and the appropriate punching hole radius for engineering calculations has not been thoroughly studied.

Building upon the literature survey conducted above, this study aims to perform a numerical analysis of explosion shock wave propagation and its interaction with the RC slab. The focus lies in obtaining a calculation method for determining the explosion load exerted on the surface of the RC slab, as well as determining the critical explosion load necessary to induce punching shear failure. Subsequently, a calculation model is developed to estimate the punching radius of the RC slab, utilizing the Twin Shear Stress Yield Criterion (TSSYC) and the virtual work principle. To validate the model’s accuracy, it is compared with existing test results.

## 2. Explosion Load

The precise definition of explosion loading is crucial for analyzing the dynamic response and damage modes of a structure. One commonly utilized method to describe explosion shock waves is through overpressure formulas [32]. These formulas establish a relationship between the weight of the TNT charge, denoted as W, and the distance between the explosive and the target, referred to as R. These parameters are often expressed in terms of the scaled distance, Z, which acts as a scaling parameter ensuring that different TNT charge weights result in equivalent blast overpressure at the same scaled distance. The scaling law, as defined by Hopkinson, is as follows:

(1) $$Z=\frac{R}{W^{1/3}}$$

**Figure 1:**
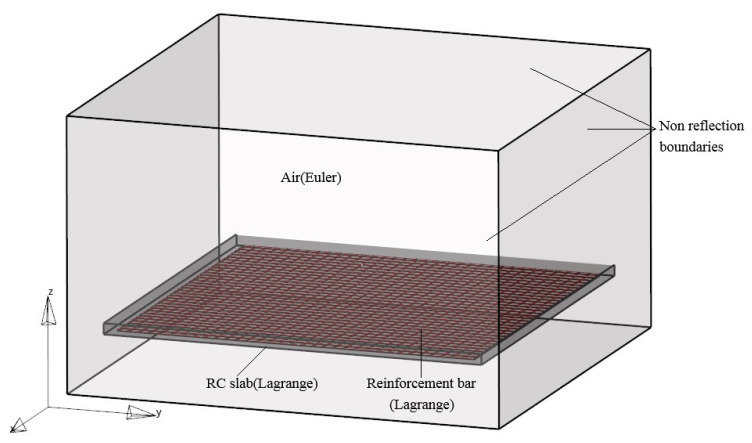
The three-dimensional finite element model.

The commonly employed peak-reflected overpressure formulas for explosion shock waves, developed based on infinite reflection surface conditions derived from explosion test results, are not applicable to structure members with finite dimensions. In this section, a three-dimensional fully coupled finite element model is constructed using Autodyn. The model includes the TNT charge, air, and an RC slab, enabling the study of explosion load distribution on finite-size plates, and the displacement and rotation of the four-edge of the RC slab are limited. The RC slab has dimensions of 6 m in length and width, with a thickness of 0.1 m. The steel reinforcement has a diameter of 10 mm. The TNT explosive is represented as a spherical charge positioned directly above the center of the RC slab. By varying the weight of the TNT charge and the distance between the charge and the RC slab, explosion shock waves with different scaled distances are obtained. The three-dimensional finite element model is depicted in Figure 1. 

In the simulation, the Euler algorithm is employed for the air domain and TNT explosive, while the Lagrange algorithm is used for the concrete and steel reinforcement. The fluid-solid coupling method is utilized to capture the interaction between the explosion shock wave and the RC slab. The concrete and reinforcement are connected through shared nodes. The 1/4 symmetry modeling method is applied to reduce computational time, taking advantage of the structure’s symmetry. Flow-out boundaries are implemented on the external surfaces of the air domain to simulate an infinite fluid medium. The element size plays a crucial role in the accuracy of the numerical simulation results. In a study by Kuang [33], the size of the fluid mesh was found to be proportional to the cube root of the charge weight. For scaled distances ranging from 0.2 m/kg^1/3^ to 0.5 m/kg^1/3^, it is recommended to use a fluid mesh size equivalent to 2.5 to 5 times the cube root of the charge mass. In this study, an 8.3 mm fluid mesh size is adopted to ensure accuracy and reliability in simulating the explosion load. For better simulation of the fluid-solid interaction, the Euler mesh size should be close to the Lagrange mesh size in the fluid-solid coupling calculation. Therefore, a Lagrange mesh size of 5 mm is chosen.

**Figure 2:**
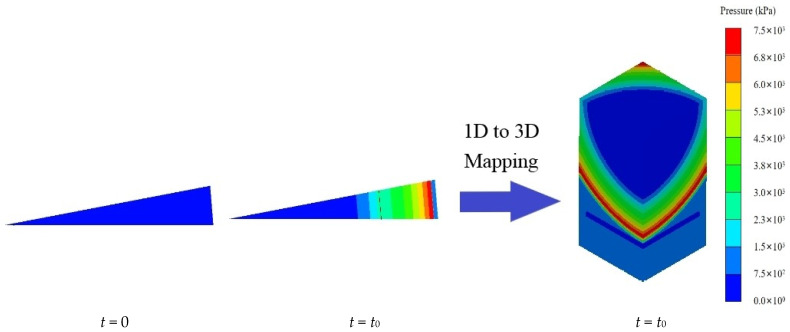
One-dimensional model results mapped onto that of the three-dimensional model.

In this study, a one-dimensional to three-dimensional mapping method is utilized to apply the explosion load to the three-dimensional model, as illustrated in Figure 2. This method enhances the analysis efficiency of the model and reduces computational time. Initially, in the one-dimensional axisymmetric model, the process leading up to the moment just before the explosion shock wave reaches the structure is solved. Subsequently, the mapping algorithm is employed to transfer the pressure of the explosion shock wave, material distribution, and other relevant information onto the three-dimensional model. The explosion shock wave continues to propagate and interact with the structure until the end of the calculation time. This approach allows efficient and accurate simulation of the explosion’s effects on the three-dimensional structure.

### 2.1. Material Model

The model for air in the simulation utilizes the ideal gas equation of state. This equation describes the relationship between pressure, volume, and temperature for an ideal gas. In the context of the simulation, the relationship between pressure and specific internal energy (energy per unit mass) is expressed as follows:

(2) p=(γ−1)ρe

where ρ is the density of air, e is the initial internal energy of air, and γ is the material constant. The ideal gas equation of state parameters are listed in Table 1.

During the explosion, the TNT charge undergoes a rapid transformation from a concentrated solid to a high-temperature and high-pressure gas. To accurately capture the process of explosive energy release, the Jones–Wilkins–Lee Equation of State (JWL EOS) is utilized, which is given via:

(3) $$P=A\left(1-\frac{\omega }{R_{1}V}\right)e^{-R_{1}V}+B\left(1-\frac{\omega }{R_{2}V}\right)e^{-R_{2}V}+\frac{\omega E}{V}$$

where P is the overpressure of the explosion product, V is the relative volume of the explosion product, and E is the initial internal energy of the explosion product. *A*, *B*, *R*_1_, *R*_2_, and ω are the parameters of the EOS. The JWL EOS effectively describes the behavior of high explosives and provides a comprehensive representation of the energy release process. It accounts for factors such as the detonation velocity, the expanding gas volume, and the energy released during the explosion. By incorporating the JWL EOS, the simulation can accurately model the dynamic behavior and energy release characteristics of the TNT charge during the explosion.

As can be seen, three terms are contained in the JWL EOS given using Equation (3). Based on the experiments and numerical simulation, it can be known that the three stages of high pressure, medium pressure, and low pressure of detonation products are controlled by the first, second, and third terms in the JWL EOS, respectively. In contrast, the third term has a more significant effect on the low voltage range. The parameters of the JWL EOS are listed in Table 2.

Under high-speed loads, concrete materials exhibit significant strain rate strengthening effects. This means that the compressive strength and tensile strength of concrete are significantly increased when subjected to high strain rates. To capture these effects, the Riedel–Hiermaier–Thoma (RHT) dynamic damage model is employed to simulate the behavior of concrete in the present study. The RHT model [34] is derived from the Holmquist–Johnson–Cook model and is specifically designed to accurately simulate the failure and dynamic response of brittle materials such as concrete and rock under dynamic loading conditions. The model considers the influence of the third invariant J3 of the deviator stress tensor on the shape of the failure surface. The RHT model has been widely applied in numerical simulations of various scenarios involving explosions, impacts, penetrations, and other dynamic events. By utilizing the RHT model, the simulation can effectively capture the dynamic behavior and failure characteristics of concrete under the explosion loading conditions considered in this study.

In the RHT model, there are three key equations: the elastic limit surface equation, the failure surface equation, and the residual failure surface equation. These equations govern the behavior of concrete and capture its elastic and failure characteristics. The failure surface equation represents the relationship between the pressure p*, Lode angle *θ*, and strain rate. It is defined as follows:

(4) $$Y_{fail}\left(p*,\theta ,\dot{\varepsilon }\right)=Y_{c}\left(p*\right)\cdot R\left(\theta \right)\cdot F_{rate}\left(\dot{\varepsilon }\right)$$

where *Y_c_*(p*) is the compressed meridian, *R*(*θ*) is the function of the Lode angle, *F_rate_*($\dot{\varepsilon }$) is the enhancement factor of strain rate. The elastic limit surface equation is defined as follows:

(5) $$Y_{e}=Y_{fail}\cdot F_{e}\cdot F_{CAP}\left(p*\right)$$

where *F_e_* is the ratio of elastic strength to failure strength and *F_CAP_*(p*) is the elastic limit cap function which is used to limit the elastic deviatoric stress under hydrostatic pressure, and the expression is as follows:

(6) $$F_{CAP}\left(p*\right)=\left\{\begin{array}{ll} 1 & p*\leq p_{u}\\ \sqrt{1-{\left(\frac{p*-p_{u}}{p_{0}-p_{u}}\right)}^{2}} & p_{u}<p*<p_{0}\\ 0 & p*\geq p_{0} \end{array}\right.$$

where *p*_0_ equals the current pore crush pressure, *p_u_* equals a third of the compressive strength. The residual failure surface equation is defined as follows:

(7) $$Y_{residual}^{*}=B\cdot {\left(p*\right)}^{M}$$

where *B* is the residual failure surface constant and *M* is the residual failure surface index. For more details of the RHT model, see Reference [34]. The input parameters of the RHT model are listed in Table 3.

Under high-speed loads, steel reinforcement also exhibits a significant strain rate strengthening effect, leading to a substantial increase in strength. To accurately capture this behavior, the Johnson–Cook model is utilized to simulate the mechanical properties of steel reinforcement under high strain rates and elevated temperatures. The flow stress in the Johnson–Cook model is expressed as follows:

(8) $$\sigma_{y}=\left(A+B\varepsilon_{p}^{n}\right)\left(1+C\ln\frac{{\dot{\varepsilon }}_{p}}{{\dot{\varepsilon }}_{0}}\right)\left(1-T^{m}\right)$$

where *A* is the yield strength of the material, ε_p_ is the plastic strain, *B* and *n* are the hardening constants of the strain rate, respectively, *C* is the strain rate sensitivity coefficient, ${\dot{\varepsilon }}_{p}$ is the plastic strain rate, ${\dot{\varepsilon }}_{0}$ is the reference plastic strain rate, *T* is the homologous temperature, and *m* is the thermal softening exponent. The input parameters of the Johnson–Cook model are listed in Table 4.

### 2.2. Validation of Explosive Pressure

**Figure 3:**
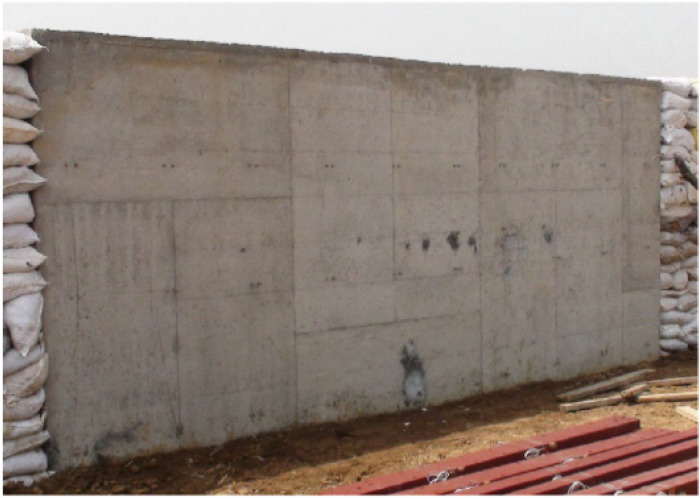
Test set-up [35].

To validate the reliability and accuracy of the numerical simulation method for the propagation and interaction of explosion shock waves with the structure slab, a simulation is conducted to replicate an explosion test performed by Nian [35]. The RC structure is 6 m long, 0.4 m thick, and 2.5 m high, and the displacement and rotation of the one-edge of the RC structure are limited. An amount of 20 kg of TNT explosive is placed on the ground and on the central axis of the wall at 3 m. The sensor for the test of reflective pressure is fixed on the central axis of the structure at a distance of 0.22 m from the ground. The test configuration and the structure’s geometry are depicted in Figure 3.

**Figure 4:**
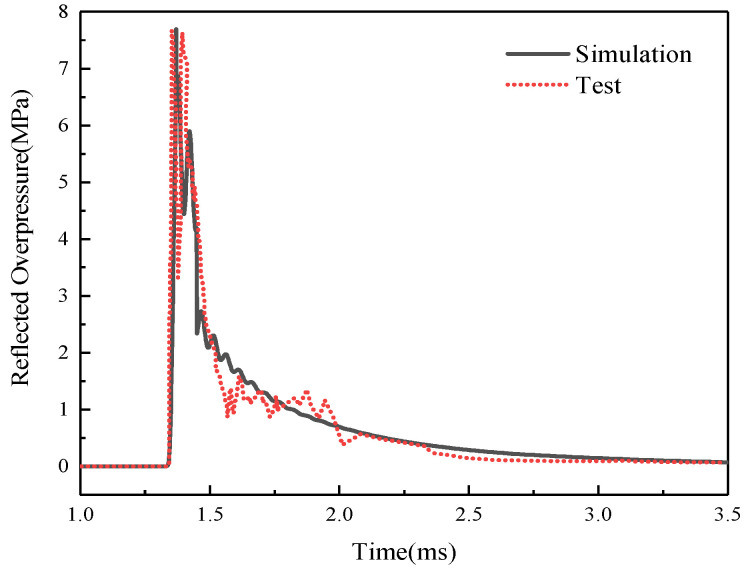
Reflection overpressure comparison.

The comparison between the numerical simulation results and the test data for the reflected pressure of the explosion shock wave, expressed in pg-1, is illustrated in Figure 4. It demonstrates a generally favorable agreement between the numerical and test results. This agreement indicates that the proposed numerical simulation method presented in this study is effective in predicting the reflected pressure on RC slabs.

### 2.3. Explosion Load on the Surface of RC Slabs

**Figure 5:**
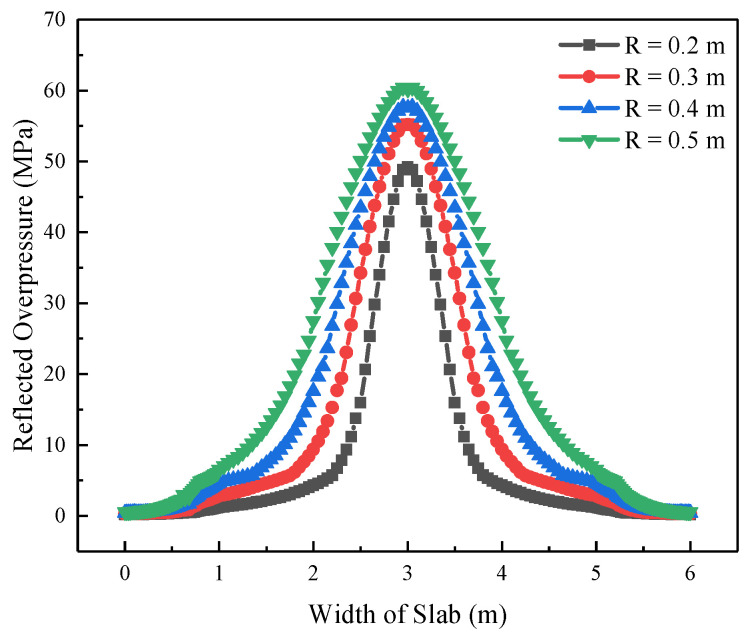
Distribution of peak reflected overpressure on the RC slab.

Sixteen numerical models were built with scaled distances of 0.2, 0.3, 0.4, and 0.5 m/kg^1/3^ and explosion distances of 0.2, 0.3, 0.4, and 0.5 m, respectively. Figure 5 shows the distribution of the peak reflected overpressure on the RC slab along the width with different explosion distances when the scaled distance is 0.3 m/kg^1/3^. It reveals that the distribution of explosion load on the front surface of the RC slab is non-uniform. With the increase of distance from the target point to the center of the RC slab, the peak reflected overpressure decreases exponentially. Moreover, when the scaled distance is the same, the explosion load gradually increases with an increase in the explosion distance.

To provide a more intuitive analysis of the peak reflected overpressure, *P_r_*, on RC slabs, two parameters are introduced: the amplification coefficient of the peak reflected overpressure, *A_p_*, and the normalization coefficient, *ξ_p_*. *A_p_* represents the ratio of the peak reflected overpressure to the peak incident overpressure, while *ξ_p_* represents the ratio of the peak reflected overpressure at a distance of *r* from the center of the slab to the peak reflected overpressure at the center.

In this study, the peak incident overpressure, *P_i_*, given by Equation (9), is estimated using the relationship proposed by Baker [36], which is based on experimental data from spherical blast tests. The fitting formulas for *A_p_*, *ξ_p_*, and the positive overpressure duration, *t_f_*, given using Equations (10)–(12), are obtained through the least squares curve fitting method applied to the numerical simulation results. These formulas allow for a comprehensive characterization of the peak reflected overpressure and its distribution across the RC slabs.

(9) $$P_{i}=\left\{\begin{array}{c} \frac{2.006}{Z}+\frac{0.194}{Z^{2}}-\frac{0.004}{Z^{3}},\;0.05\;m/{\mathrm{kg}}^{1/3}\leq Z\leq 0.5\;m/{\mathrm{kg}}^{1/3}\\ \frac{0.067}{Z}+\frac{0.301}{Z^{2}}+\frac{0.431}{Z^{3}},\;0.5\;m/{\mathrm{kg}}^{1/3}\leq Z\leq 70.9\;m/{\mathrm{kg}}^{1/3} \end{array}\right.$$

(10) $$A_{p}=4.656+39.442\exp\left(-10.723ZW^{-1/15}\right)$$

(11) $$\xi_{p}=\exp\left(-1.109r/R\right)$$

(12) $$t_{f}=W^{1/3}\left[0.437+0.004\exp\left(4.950Z\right)\right]$$

The fitting Formulas (10)–(12) exhibit regression coefficients of 0.992, 0.981, and 0.991, respectively. These high coefficients indicate a strong agreement between the fitting formulas and the numerical data, validating the accuracy of the formulas.

To analyze the explosion shock wave’s reflected overpressure, an ideal inverted triangle model is adopted to simplify the load applied to the RC slab. The expression of the explosion load is as follows:

(13) $$P_{r}=A_{p}P_{i}\xi_{p}\left(1-t/t_{f}\right),\;0.2\;m/{\mathrm{kg}}^{1/3}\leq Z\leq 0.5\;m/{\mathrm{kg}}^{1/3}$$

For different explosive materials, the corresponding TNT equivalent can be converted and substituted into the above formulae for calculation. The expression of the equivalent conversion of explosion energy is as follows:

(14) $$WQ_{TNT}=W_{e}Q_{e}$$

where *W* is the mass of TNT, *Q*_TNT_ is the explosion heat of TNT, *W_e_* is the mass of other explosion materials, and *Q_e_* is the explosion heat of other explosion materials.

## 3. Punching Shear Failure Analysis

**Figure 6:**
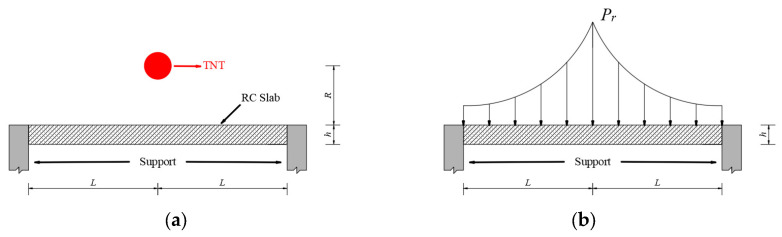
(**a**) Physical model; (**b**) load model.

In real buildings, RC slabs are usually cast monolithically with beams, which can enhance significantly their bearing capacity [37]. Therefore, when analyzing the response of the RC slab to the explosion load, the structure shown in Figure 6 is selected as the calculation model. It is assumed that a spherical TNT charge with a weight of W detonates at a distance of R from the surface of the square RC slab. It is observed that under an intense dynamic load, the energy absorbed by the plastic deformation of the RC slab surpasses that absorbed by elastic deformation. To simplify the analysis, the failure of the RC slab under the explosion load can be studied using the ideal rigid plastic model.

### 3.1. Critical Punching Shear Failure Load

The extent of damage to RC slabs is closely associated with the maximum dynamic response, which is typically estimated via the support rotation angle. Previous research has indicated that punching damage occurs when the support rotation angle of the RC slab exceeds 6.8° [12]. Therefore, in this study, punching shear failure is defined when the support rotation angle of the RC slab is greater than 6.8°.

**Figure 7:**
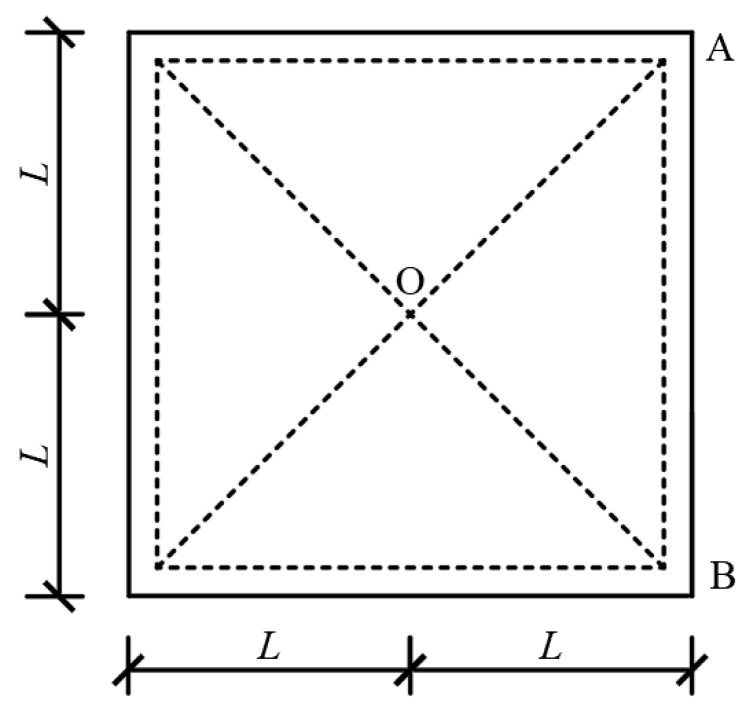
Damage mechanism and analysis model.

The concrete material is assumed to exhibit ideal plastic behavior, and the deformation of the slab is concentrated along the plastic hinge lines (OA, OB), as depicted in Figure 7. The position of the plastic hinge lines is assumed to remain constant throughout the loading process. Due to the slab’s symmetry and the applied load, a 1/4 symmetry structure is employed as the analysis model.

The mid-span displacement is set as *u*_0_. The rotation of plate OAB is rotated around the AB axis, and the rotation angle, β, can be represented as follows:

(15) $$\beta =\tan\beta =\frac{u_{0}}{L}$$

The displacement, velocity, and acceleration of plate OAB can be represented as follows:

(16) $$u=\frac{L-x}{L}u_{0},\dot{u}=\frac{L-x}{L}{\dot{u}}_{0},\ddot{u}=\frac{L-x}{L}{\ddot{u}}_{0}$$

In the analysis model, the total strain energy is calculated as the sum of the plastic strain energy of the concrete slab based on the assumption that the slab material behaves plastically. The previous test results show that the RC members always exhibit local damage with little overall damage under the close-in explosion load [11]. The strain of steel reinforcement in the punching zone is typically small and undergoes minimal yielding. Moreover, the volume of steel reinforcement is relatively smaller compared to concrete. Therefore, the contribution of steel reinforcement strain is not considered in the calculation of plastic energy.

The bending capacity of the slab unit width can be defined as the moment the slab can resist before failure occurs. It is influenced by various factors, including the properties of the steel reinforcement and the compressive strength of the concrete. Assuming a uniformly arranged steel reinforcement with a cross-sectional area of reinforcement unit width of *A_s_* and a reinforcement ratio of *ρ_s_*, and considering the dynamic tensile strength of the steel reinforcement as *f_yd_* and the dynamic compressive strength of concrete as *f_cd_*, the bending capacity can be expressed as:

(17) $$m=A_{s}f_{yd}h_{0}\left(1-0.5\rho_{s}\frac{f_{yd}}{f_{cd}}\right)$$

where *h*_0_ is the effective height of the slab section.

The total strain energy of the analysis model can thus be written as:

(18) $$E=\sqrt{2}Lm_{OA}\beta +\sqrt{2}Lm_{OB}\beta$$

The strain energy of the analysis model is obtained by substituting Equation (17) into Equation (18) as follows:

(19) $$E=2\sqrt{2}LA_{s}f_{yd}h_{0}\left(1-0.5\rho_{s}\frac{f_{yd}}{f_{cmd}}\right)u_{0}$$

The kinetic energy of the analysis model is defined as:

(20) $$T=\frac{1}{2}∯\rho h{\dot{u}}^{2}dS$$

The force on the surface of the RC slab can be written as:

(21) $$Q=∯P_{r}dS$$

Neglecting the influence of damping during the movement of the RC slab, the Lagrange equation for the analysis model is established as follows:

(22) $$\frac{d}{dt}\left(\frac{\partial T}{\partial \dot{u}}\right)-\frac{\partial T}{\partial u}+\frac{\partial E}{\partial u}=Q$$

The acceleration of the RC slab is:

(23) $${\ddot{u}}_{0}=\frac{Q}{\eta }\left(1-\frac{t}{t_{f}}\right)-\frac{E}{\eta }$$

where

(24) $$\left\{\begin{array}{l} Q=\frac{\pi }{2}P_{r}R\left[1-\left(1+1.109\frac{L}{R}\right)\exp\left(-\frac{1.109L}{R}\right)\right]\\ E=2\sqrt{2}LA_{s}f_{yd}h_{0}\left(1-0.5\rho_{s}\frac{f_{yd}}{f_{cmd}}\right)\\ \eta =\frac{\rho hL^{2}}{3} \end{array}\right.$$

The initial conditions of Equation (23) are:

(25) $$u_{0}\left(0\right)={\dot{u}}_{0}\left(0\right)=0$$

The expressions for the velocity and the displacement of slab OAB are obtained as follows:

(26) $$\left\{\begin{array}{l} {\dot{u}}_{0}=\frac{Q}{\eta }\left(t-\frac{t^{2}}{2t_{f}}\right)-\frac{E}{\eta }t\\ u_{0}=\frac{Q}{\eta }\left(t^{2}-\frac{t^{3}}{6t_{f}}\right)-\frac{Et^{2}}{2\eta } \end{array}\right.$$

When the time reaches *t_f_*, the mid-span of the slab reaches its maximum displacement, which can be written as:

(27) $$u_{0,\max}=\frac{t_{f}^{2}}{6\eta }\left(2Q-3E\right)$$

According to the criteria for determining the punching shear failure of RC slabs, the expression for the critical explosion load can be obtained as:

(28) $$P_{r,dyn}=\frac{3Et_{f}^{2}+6\eta L\tan6.8{}^{\circ}}{\pi Rt_{f}^{2}\left[1-\left(1+1.109L/R\right)\exp\left(-1.109L/R\right)\right]},\;0.2\;m/kg^{1/3}\leq Z\leq 0.5\;m/kg^{1/3}$$

### 3.2. Radius of Punching

**Figure 8:**
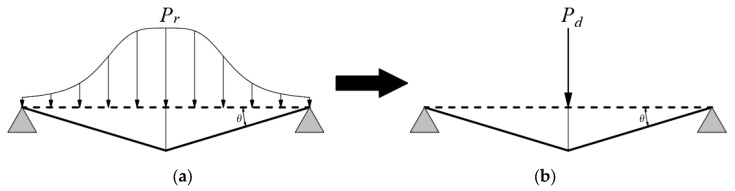
(**a**) Deformation under explosion load; (**b**) deformation under concentrated load.

The deformation mechanism of the square RC slab under the non-uniform explosion load and the dynamic concentrated load is shown in Figure 8. To analyze the punching shear failure of the RC slab, the non-uniform explosion load is transformed into a dynamic concentrated load using the principle of equal virtual work. This conversion is based on the work-energy principle, which states that the work done by a load is equal to the change in the strain energy of the structure.

The program developed by Lu and Silva [13] allows for the conversion of the non-uniform explosion load into a dynamically concentrated load that can be incorporated into the analysis. Considering the principle of virtual work, the concentrated load is determined to produce the same deformation and strain energy as the non-uniform explosion load. This approach enables the analysis of the punching shear failure of the RC slab under the simplified dynamic concentrated load, which facilitates the evaluation of the structural response and the determination of the critical punching radius.

The load and the deformation of the RC slab are symmetrically distributed, and the rotation angle of the RC slab support at any time is *θ*. The virtual work done by the dynamic concentrated load *P_d_* is:

(29) $$W_{e}=P_{d}L\theta$$

The virtual work done by the non-uniform explosion load *P_r_* is:

(30) $$W_{p}=4\theta \int_{0}^{L}dy\int_{0}^{L}P_{r}\left(L-x\right)dx$$

Equating Equation (29) to Equation (30), the equivalent dynamic concentrated load can be obtained as:

(31) $$P_{d}=\frac{4P_{r}\kappa }{L}$$

where *κ* is the load transformation factor computed based on the relation:

(32) $$\kappa =\int_{0}^{L}dy\int_{0}^{L}\left(L-x\right)dx$$

According to the TSSYC proposed by Yu [38], which considers the influence of three principal stresses and compensates for the lack of consideration of the intermediate principal stress in the Mohr–Coulomb strength theory. Assuming that the tensile strength of concrete is *f_td_* and the compressive strength is *f_cd_* and $\bar{m}=f_{td}/f_{cd}$, the failure condition of the concrete in the context of punching shear failure of the RC slab can be expressed as follows:

(33) $$\sigma_{1}-\frac{1}{2\bar{m}}\left(\sigma_{2}+\sigma_{3}\right)=0$$

Due to the symmetry of the displacement field and that the plastic deformation is assumed to be small and incompressible, the intermediate principal stress can be written as:

(34) $$\sigma_{2}=\frac{1}{2}\left(\sigma_{1}+\sigma_{3}\right)$$

Substituting Equation (34) into Equation (33), the twin shear stress yield condition under axisymmetric working conditions can be obtained as follows:

(35) $$K_{1}\sigma_{1}-K_{2}\sigma_{3}=f_{td}$$

where *K*_1_ and *K*_2_ are given by:

(36) $$K_{1}=1-1/4\bar{m},\;K_{2}=3/4\bar{m}$$

Assuming the normal stress on the failure surface is $\sigma_{n}$ and the shear stress is $\tau_{r}$, the ultimate stress circle equation is given by:

(37) $$G={\left(\sigma_{n}-\frac{\sigma_{1}+\sigma_{3}}{2}\right)}^{2}+\tau_{r}^{2}-{\left(\frac{\sigma_{1}-\sigma_{3}}{2}\right)}^{2}=0$$

Substituting Equation (35) into Equation (37) and eliminating σ_1_, the ultimate stress circle equation is rewritten as:

(38) $$G={\left(\sigma_{n}-\frac{f_{td}+\left(K_{1}+K_{2}\right)\sigma_{3}}{2K_{1}}\right)}^{2}+\tau_{r}^{2}-{\left(\frac{f_{td}+\left(K_{1}-K_{2}\right)\sigma_{3}}{2K_{1}}\right)}^{2}=0$$

When the value of $\partial G/\partial \sigma_{3}$ is 0 and eliminating *σ*_3_, the envelope equation of the limit stress circle can be obtained as:

(39) $$\left(K_{1}-K_{2}\right)\sigma_{n}\pm 2\sqrt{K_{1}K_{2}}\tau_{r}-f_{td}=0$$

According to Equation (39), the angle *θ* between the envelope and *X*-axis is:

(40) $$\tan\theta =\frac{K_{1}-K_{2}}{2\sqrt{K_{1}K_{2}}}$$

**Figure 9:**
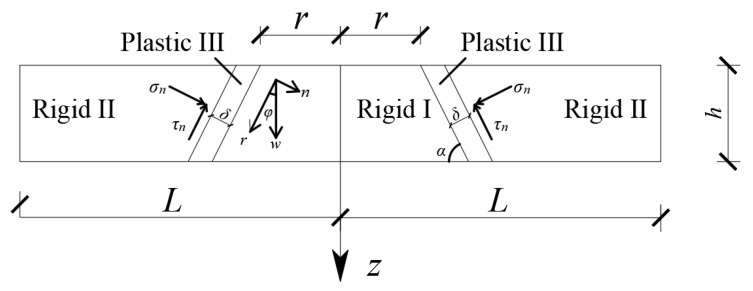
The punching model of the RC slab.

In the analysis of punching shear failure in the RC slab, the punching shear failure surface is assumed to be a truncated cone surface with a straight line as the generatrix. The RC slab is divided into three regions, rigid I, rigid II, and plastic III, as shown in Figure 9. The initial thickness of the plastic zone is denoted as *δ*. The normal direction of the generating line is represented by *n*, and the tangent direction is represented by *r*. The angle between the generating line and the tangent direction is denoted as *φ*. The overall thickness of the RC slab is *h*. This assumption allows for the characterization of the deformation and failure behavior of the RC slab during punching. The plastic zone represents the region where significant deformation and failure occur, while the rigid regions on either side of the plastic zone remain relatively undeformed. By considering the geometry and material behavior in this manner, the analysis can focus on the critical area where punching shear failure is likely to occur and accurately capture the response of the RC slab under the applied load.

When rigid I generates a vertical virtual displacement, *w*, relative to rigid II, the strain and strain rate in the plastic zone become:

(41) $$\varepsilon_{n}=\frac{w\sin\phi }{\delta },\;\varepsilon_{r}=\frac{w\cos\phi }{\delta }$$

(42) $${\dot{\varepsilon }}_{n}=\frac{\dot{w}\sin\phi }{\delta },\;{\dot{\varepsilon }}_{r}=\frac{\dot{w}\cos\phi }{\delta }$$

According to Equation (42), the expression of the angle between the generating line and the tangent direction is:

(43) $$\tan\phi =\frac{{\dot{\varepsilon }}_{n}}{{\dot{\varepsilon }}_{r}}$$

According to the associated flow rule of plastic theory, it can be concluded that:

(44) $$\frac{{\dot{\varepsilon }}_{n}}{{\dot{\varepsilon }}_{r}}=-\frac{d\tau_{r}}{d\sigma_{n}}=\tan\theta$$

The value of the angle between the generating line and the tangent direction can be obtained using Equations (43) and (44) as:

(45) θ=ϕ

Due to the axial symmetry of the velocity field, the circumferential strain is 0. According to the plastic theory, the linear strain along the sliding line is 0. Therefore, the plastic energy consumed by plastic III of concrete at any time during the punching process is:

(46) $$W_{p}=∯\left(\sigma_{n}\varepsilon_{n}+\tau_{r}\varepsilon_{r}\right)\delta \;dS$$

In Equation (46), *dS* is the area of the damaged cone surface given by:

(47) $$dS=2\pi \left(r+z\tan\phi \right)\frac{dz}{\cos\phi }$$

The plastic energy can be obtained from Equations (41), (46), and (47) as:

(48) $$W_{p}=\frac{\pi h^{2}f_{td}w}{\sqrt{K_{1}K_{2}}}\left(\frac{r}{h}+\frac{K_{1}-K_{2}}{4\sqrt{K_{1}K_{2}}}\right)$$

According to the equation between the virtual work by the dynamic concentrated load and the plastic energy consumed in the plastic zone, the radius of the punching hole can be obtained as:

(49) $$r=\frac{4\delta \sqrt{K_{1}K_{2}}}{\pi hLf_{td}}P_{r}-\frac{K_{1}-K_{2}}{4\sqrt{K_{1}K_{2}}}h,\;0.2\leq Z\leq 0.5$$

## 4. Test Verification

Based on the comparison between the calculation results and the experimental results presented in Table 5, it can be observed that the proposed approach accurately predicts the punching failure of the RC slab. By contrasting the calculated results obtained from Equation (13) with those from Equation (28), it is possible to determine whether the RC slab will experience punching shear failure or not. Additionally, the radius of the punching hole obtained using Equation (49) shows good agreement with the estimated experimental results. This further supports the effectiveness of the method that applies the TSSYC and the virtual work principle to predict the punching shear failure of RC slabs. Therefore, based on the comparison and agreement between the theoretical analysis results and the experimental data, it can be concluded that the proposed approach is accurate and reliable in predicting the punching shear failure of RC slabs.

## 5. Conclusions

In conclusion, the present study successfully investigated the dynamic response and punching shear failure characteristics of RC slabs subjected to close-in explosion loads. The reliability of the numerical simulation was verified through comparison with experimental data, and a fitting formula for the close-in explosion load on RC slabs was derived based on the numerical results. The punching shear failure of RC slabs under close-in explosion loads was analyzed using the TSSYC and the virtual work principle, and the contribution of steel reinforcement strain was not considered in the calculation of plastic energy. The analysis results showed good agreement with experimental data. The key conclusions of the study are as follows:

(1) The numerical method accurately predicts the distribution of reflected overpressure on RC slabs under close-in explosions, as demonstrated by the agreement with experimental results.
(2) The proposed approach based on the TSSYC and the principle of virtual work accurately predicts the punching hole radius of RC slabs under close-in explosions, with theoretical calculations aligning well with experimental observations.
(3) The scaled distance, slab thickness, and concrete strength are significant factors influencing the punching hole radius of RC slabs under close-in explosions.

These findings contribute to a better understanding of the dynamic behavior and failure mechanisms of RC slabs subjected to close-in explosion loads. The results can be utilized in the design and assessment of structures to enhance their resistance against blast effects.

## Figures and Tables

**Table 1:** Parameters for the air material.

*ρ* (kg/m^3^)	*e* (kJ/kg)	*γ*
1.225	2.068 × 105	1.4

**Table 2:** Parameters for TNT charge and JWL EOS.

*A* (MPa)	*B* (MPa)	*R*_1_	*R*_2_	*ω*	*ρ* (kg⋅m^3^)	*D* (m/s)	*E* (MPa)	*P_CJ_* (MPa)
3.712 × 10^5^	3.231 × 10^3^	4.15	0.95	0.30	1.630	6930	7 × 10^3^	2.1 × 10^4^

**Table 3:** Parameters for the RHT model.

*ρ* (kg⋅m^−3^)	*G* (MPa)	*f_c_* (MPa)	*p_el_* (MPa)	*f_t_*/*f_c_*	*f_s_*/*f_c_*	*A*
2.75 × 10^3^	1.7 × 10^4^	40	23.3	0.1	0.18	1.6
***N***	***ε_f_*_,_ *_min_***	***B***	***M***	***D*_1_**	***D*_2_**	***Erosion***
0.61	0.01	0.7	0.53	0.04	1	0.01

**Table 4:** Parameters for the Johnson–Cook model.

*ρ* (kg⋅m^3^)	*v*	*A* (MPa)	*B* (MPa)	*n*	*C*	${\dot{\varepsilon }}_{0}$	*m*
7.83 × 10^3^	0.26	300	384	0.26	0.014	1	1

**Table 5:** Comparison between experimental data and theoretical analysis results.

Test	Size (mm)	*W* (kg)	*R* (m)	Punching Observed	Test Result (mm)	*P_r_* (MPa)	*P_r_*_,_*_dyn_* (MPa)	Calculated Result (mm)	Deviation
Wang et al. (2012) [8]; *f_c_* = 39.5 MPa, *f_y_* = 600 MPa									
A	750 × 750 × 30	0.13	0.3	No	—	19.00	80.46	—	—
B	750 × 750 × 30	0.19	0.3	No	—	26.61	68.24	—	—
C	1000 × 1000 × 40	0.31	0.4	No	—	15.92	45.20	—	—
D	1000 × 1000 × 40	0.46	0.4	No	—	22.60	38.03	—	—
E	1250 × 1250 × 50	0.64	0.5	No	—	15.57	22.68	—	—
F	1250 × 1250 × 50	0.94	0.5	Yes	105	21.92	19.08	113	9.52%
Feng et al. (2017) [9]; *f_c_* = 30 MPa, *f_y_* = 650 MPa									
B1-1	1100 × 1000 × 40	0.2	0.4	No	—	10.85	51.43	—	—
B1-2	1100 × 1000 × 40	0.4	0.4	No	—	19.95	40.65	—	—
B1-3	1100 × 1000 × 40	0.6	0.4	No	—	26.27	33.27	—	—
B2-1	1100 × 1000 × 40	0.2	0.4	No	—	10.85	51.40	—	—
B2-2	1100 × 1000 × 40	0.4	0.4	No	—	19.95	40.62	—	—
B2-3	1100 × 1000 × 40	0.6	0.4	No	—	26.27	33.24	—	—
B3-1	1100 × 1000 × 40	0.2	0.4	No	—	10.85	51.39	—	—
B3-2	1100 × 1000 × 40	0.4	0.4	No	—	19.95	40.60	—	—
B3-3	1100 × 1000 × 40	0.6	0.4	Yes	200	26.27	33.23	231	15.50%
Shi et al. (2020) [11]; *f_c_* = 30 MPa, *f_y_* = 400 MPa									
1	2000 × 1000 × 120	2	0.4	No	—	42.19	45.55	—	—
2	2000 × 1000 × 120	2	0.4	No	—	42.19	45.55	—	—
3	2000 × 1000 × 120	4	0.4	Yes	280	55.95	29.56	327	20.36%
4	2000 × 1000 × 120	4	0.4	Yes	310	55.95	29.56	327	5.48%
5	2000 × 1000 × 120	6	0.4	Yes	400	66.21	22.88	438	9.52%
Wang et al. (2013) [12]; *f_c_* = 40 MPa, *f_y_* = 600 MPa									
I	1000 × 1000 × 40	0.2	0.4	No	—	10.85	38.55	—	—
II	1000 × 1000 × 40	0.31	0.4	No	—	15.92	33.90	—	—
III	1000 × 1000 × 40	0.46	0.4	No	—	22.60	28.53	—	—
IV	1000 × 1000 × 40	0.55	0.4	Yes	150	26.53	26.08	173	15.34%

## Data Availability

Data are contained within the article.
